# SPECT/CT Imaging of hNIS -Expression after Intravenous Delivery of an Oncolytic Adenovirus and ^131^I

**DOI:** 10.1371/journal.pone.0032871

**Published:** 2012-03-07

**Authors:** Maria Rajecki, Mirkka Sarparanta, Tanja Hakkarainen, Mikko Tenhunen, Iulia Diaconu, Venla Kuhmonen, Kalevi Kairemo, Anna Kanerva, Anu J. Airaksinen, Akseli Hemminki

**Affiliations:** 1 Cancer Gene Therapy Group, Molecular Cancer Biology Program, Haartman Institute, Transplantation Laboratory and Finnish Institute for Molecular Medicine, University of Helsinki, Helsinki, Finland; 2 HUSLAB, Helsinki University Central Hospital, Helsinki, Finland; 3 Laboratory of Radiochemistry, University of Helsinki, Helsinki, Finland; 4 Department of Oncology, Helsinki University Central Hospital, Helsinki, Finland; 5 International Comprehensive Cancer Center Docrates, Helsinki, Finland; 6 Department of Obstetrics and Gynecology, Helsinki University Central Hospital, Helsinki, Finland; University of Chicago, United States of America

## Abstract

Oncolytic adenoviruses can be engineered for better tumor selectivity, gene delivery and be armed for imaging and concentrating radionuclides into tumors for synergistic oncolysis. We constructed Ad5/3-hTERT-hNIS where replication is controlled by hTERT-promoter. Ad5/3-hTERT-hNIS expresses hNIS for imaging of transgene expression and for treatment of infected tumors by radioiodine. Ad5/3-hTERT-hNIS efficiently killed prostate cancer cells and induced iodine uptake *in vitro* and *in vivo* after intratumoral virus administration. Survival of mice treated with intravenous Ad5/3-hTERT-hNIS significantly prolonged survival over mock or radioiodine only but the combination of virus with radioiodine was not more effective than virus alone. Temporal and spatial changes in hNIS-expression during therapy were detected with SPECT, demonstrating feasibility of evaluation of the combination therapy with hNIS-expressing adenoviruses and radioiodide.

## Introduction

Gene therapy with oncolytic viruses is an emerging experimental option for treating refractory cancer lacking curative therapies. This is the case for castration resistant prostate cancer that killed estimated 27,000 men in 2009 in the U.S (http://www.cancer.gov/cancertopics/types/prostate). Oncolytic adenoviruses are promising therapeutic tools since the infection results in cytolysis of transduced cells and in the release of therapeutic progeny for further spread into target tumors. The feasibility of genetic alteration of the adenoviral genome has led to the development of several modifications that render these agents cancer selective [1], [2]. Consequently, oncolytic adenoviruses have been successfully used in clinical trials, including prostate cancer patients [3]. However, although proven safe, the efficacy of the treatments still needs to be improved. One means to achieve this is to combine them with chemotherapeutics and radiation [4], [5], [6].

Human sodium/iodide symporter (hNIS) is a transmembrane protein that is located on the basolateral membrane of thyroid cells and actively transports iodine (I^−^) for thyroid hormone synthesis. The ability to accumulate I^−^ via hNIS has provided the basis for diagnostic scintigraphic imaging in thyroid cancer and has been an effective means to target radioiodine for its treatment. The structure of oncolytic adenoviruses is compatible with the insertion of therapeutic molecules as transgenes and this feature has also been exploited for delivery of hNIS [7], [8], [9]. Recently, we and others have successfully used adenovirus-encoded hNIS to transport ^131^I into cancer cells for a combined antitumoral effect [7], [10]. In addition, hNIS-expression not only provides a therapeutic means to improve efficacy but also might serve as an imaging tool for assessing tumor size and provides information on viral kinetics during the treatment.

Recent developments in dedicated small animal multi-detector and multi-pinhole collimator scanners for single photon emission computed tomography (SPECT) have rendered the modality a powerful tool in preclinical imaging. Decades of clinical use have resulted in many commercially available tracers and kit-formulations for facile tracer radiolabeling easing the adaptation of the technique also to preclinical use. SPECT has been successfully employed in the visualization of various transgenes [11], but its advantages over other imaging modalities have rarely been more pronounced than for hNIS-imaging, where selective, readily available and inexpensive probes, ^131^I^−^, ^123^I^−^ and ^99m^TcO_4_^−^, can be employed [10].

Telomeres are structures at the ends of chromosomes consisting of hundreds to thousands of TTAGGG-repeats and associated proteins [12]. During each mitotic cell division several telomeric repeats are lost. However, some cell types, including stem and cancer cells, maintain the capacity of indefinite renewal by expressing telomerase [13], a ribonucleotide protein that can synthesize telomeres *de novo*. In fact, genetic mutations leading to constitutively active telomerase-expression have been associated with most human cancers [14]. Human telomerase reverse transcriptase (hTERT) is the catalytic subunit of human telomerase and is the major determinant of telomerase activity [15]. Since hTERT is mostly active in tumor cells, or in stem cells (not effectively killed by oncolytic adenoviruses) [16], hTERT-activity can be exploited for cancer gene therapy. This approach has been successfully used in the context of genetically modified adenoviruses to obtain tumor-specific replication not only in preclinical models [17], [18] but also in one clinical trial [19].

Gene expression by oncolytic adenoviruses is determined in part by their capability to enter target cells [20]. However, the native receptor for Ad5, coxsackie and adenovirus receptor (CAR), is often poorly expressed on cancer cells [1]. Alternative targeting strategies have been developed to overcome this and several genetic modifications of the adenoviral capsid and knob have resulted in better tumor transduction [21]. One approach includes the replacement of Ad5 knob with the serotype 3 knob since, as compared to CAR, the Ad3 receptor is expressed to a higher degree on cancer tissue and is different from CAR [22], [23], [24].

In the present study, we constructed Ad5/3-hTERT-hNIS where viral replication is controlled by the hTERT-promoter. Ad5/3-hTERT-hNIS is targeted to the Ad3 receptor for effective tumor transduction and expresses hNIS in replication-permissive cells only, thus allowing imaging of viral replication and treatment of infected tumors by radioactive iodine.

## Results

### Ad5/3-hTERT-hNIS expresses functional hNIS and is able to mediate iodine uptake into prostate cancer cells

Two new oncolytic adenoviruses, Ad5/3-hTERT-hNIS and its control virus Ad5/3-hTERT- Δgp19K, were constructed to study their antitumor effect against castration resistant prostate cancer. The Ad5 knob was replaced by the Ad3 knob to increase transduction of cancer cells [22]. The native E1A promoter was replaced by an hTERT-promoter for tumor-specific replication. In Ad5/3-hTERT-hNIS, the *gp19K* and *6.7K* in E3 is replaced by hNIS that remains under the native E3-promoter and results in replication-coupled transgene expression that starts circa 8 h post-infection [25]. The control virus harbors the same deletion (*gp19K* and *6.7K*) without insertions.

**Figure 1:**
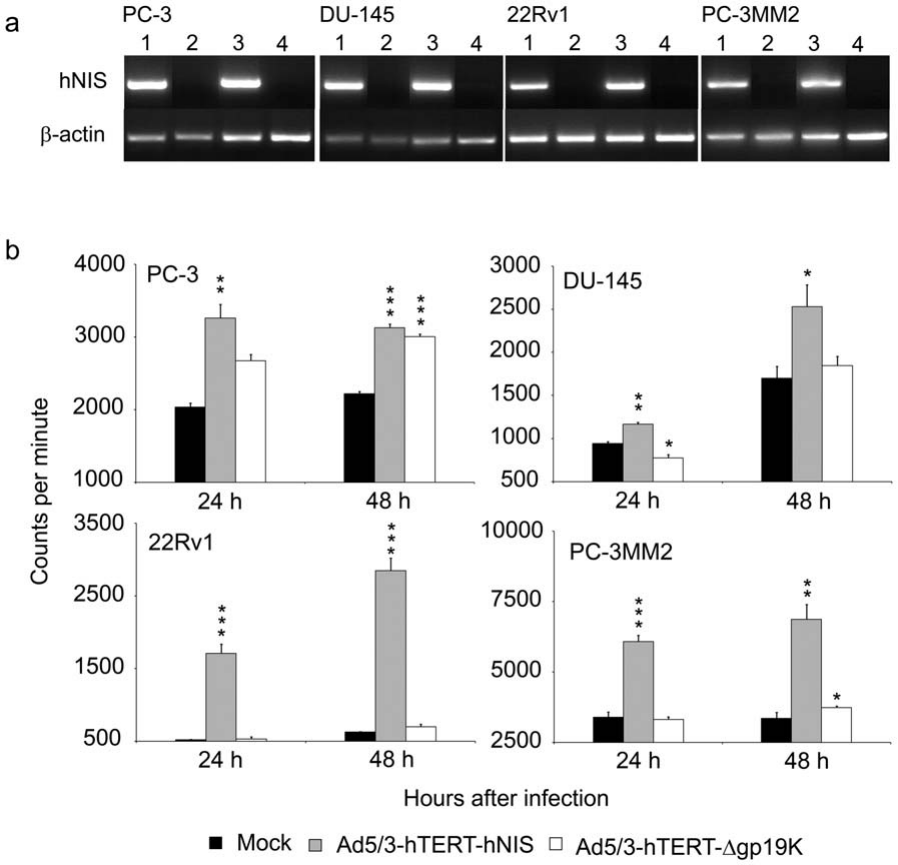
hNIS-expression in prostate cancer cells. (**a**) Cells were infected with 10 vp of Ad5/3-hTERT-hNIS (lanes 1 and 3) or control virus Ad5/3-hTERT-Δgp19K (lanes 2 and 4). hNIS-RNA -expression was assayed 24 h (lanes 1 and 2) and 48 h (lanes 3 and 4) later by RT-PCR. ß-actin served as an internal control. (**b**) ^125^I uptake in prostate cancer cells infected in triplicates with 10 vp of Ad5/3-hTERT-hNIS or Ad5/3-hTERT-Δgp19K. The capability of the cells to concentrate iodide was assessed at 24 h and 48 h after infection. Student's t-test was used for statistical analyses, *p<0.05, **p<0.01, ***p<0.001 as compared to uninfected cells. Bars represent SD.

To test the capability of the viruses to induce the expression of hNIS, castration resistant prostate cancer cells were infected with the new viruses and the cells were collected for hNIS RT-PCR at 24 h or 48 h afterwards (Figure 1a). Ad5/3-hTERT-hNIS displayed strong induction of hNIS at both time points whereas the control virus -infected cells remained negative for hNIS-expression.

Since mRNA-expression does not automatically mean production of functional hNIS, the ability of infected cells to transport and concentrate iodine was evaluated. Ad5/3-hTERT-hNIS-infected prostate cancer cells were incubated with ^125^I and then analyzed for iodide uptake using a gamma-counter (Figure 1b). 24 h after infection, iodide uptake was significantly higher in Ad5/3-hTERT-hNIS infected than in mock treated cells, p<0.001 for 22Rv1 and PC-3MM2 and p = 0.003 and 0.002 for PC-3 and DU-145 cells, respectively. Significant differences were sustained at 48 h (p<0.001 for PC-3 and 22Rv1 and 0.04 and 0.003 for DU-145 and PC-3MM2 cells, respectively). PC-3 cells seemed to accumulate some radioiodine also without virus treatment. The phenomenon has also been reported previously for other cancer types and it is known that many secretory tissues, such as the prostate, feature a weak capacity to actively transport iodide [26], [27]. This suggests that possibly hNIS-independent uptake of iodine impacted the results of the *in vitro* study.

### Ad5/3-hTERT-hNIS mediates effective oncolysis of prostate cancer cells in vitro

**Figure 2:**
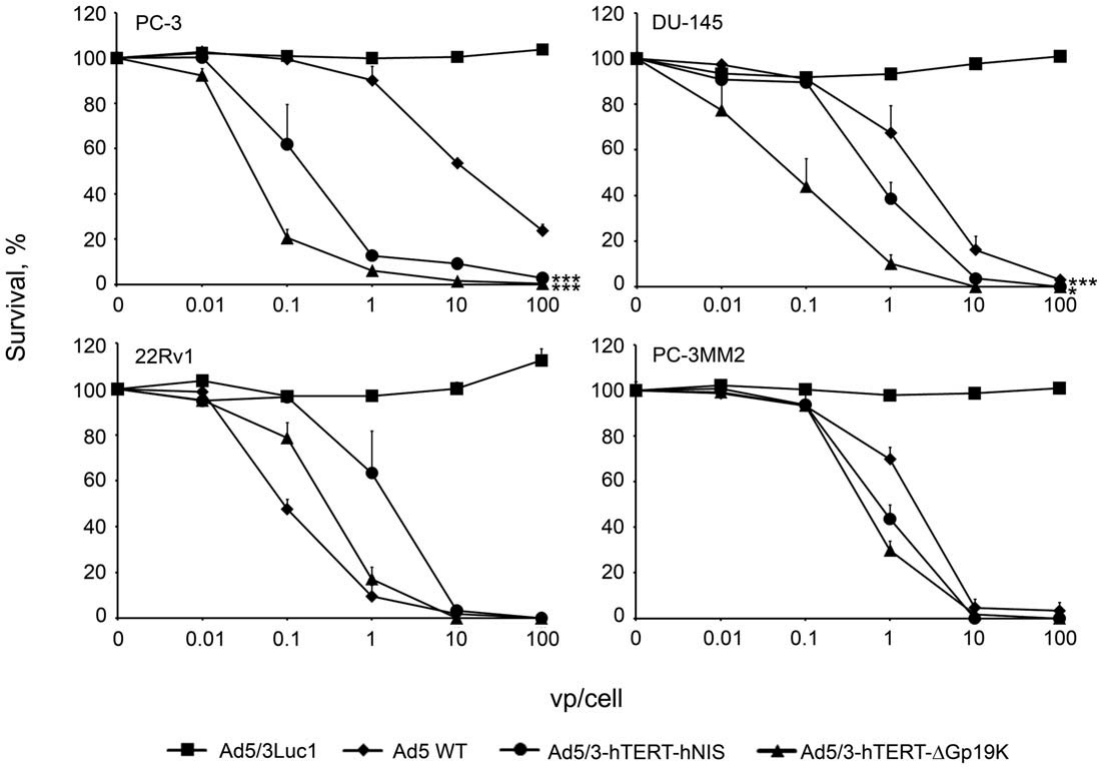
Oncolytic potency of Ad5/3-hTERT-hNIS. Prostate cancer cells were infected in triplicates with 0.01 to 100 vp/cell and the cell viability was assessed by MTS-assay. Ad5/3Luc1 is a replication-deficient adenovirus. Ad5 WT is the serotype 5 wild-type adenovirus. Ad5/3-hTERT-Δgp19K is similar to Ad5/3-hTERT-hNIS but does not contain hNIS. ***p<0.001 as compared to replication-deficient adenovirus. Bars represent SD.

To determine the oncolytic capacity of the new viruses, prostate cancer cell lines were infected with viruses and cell viability was assessed by the 3-(4,5,dimethylthiazol-2-yl)-5-(3-carboxymethoxy-phenyl)- 2-(4-sulfophenyl)-2H –tetrazolium inner salt (MTS) assay (Figure 2). Here, cell survival after infection is followed daily and the assay is stopped when the fastest virus has caused almost complete cell killing at the highest dose. In most cell lines, Ad5/3-hTERT-hNIS was more potent than wild-type adenovirus. However, some variation in the killing of different cell lines was observed, as also previously reported [24].

In the majority of the cell lines, the transgene-less Ad5/3-hTERT-Δgp19K was more efficient in prostate cancer cell killing than Ad5/3-hTERT-hNIS (Figure 2). This may be explained by the larger genome size of Ad5/3-hTERT-hNIS, caused by the transgene, which has been reported to affect oncolytic potency [7], [10].

### Iodine uptake into tumors in vivo

**Figure 3:**
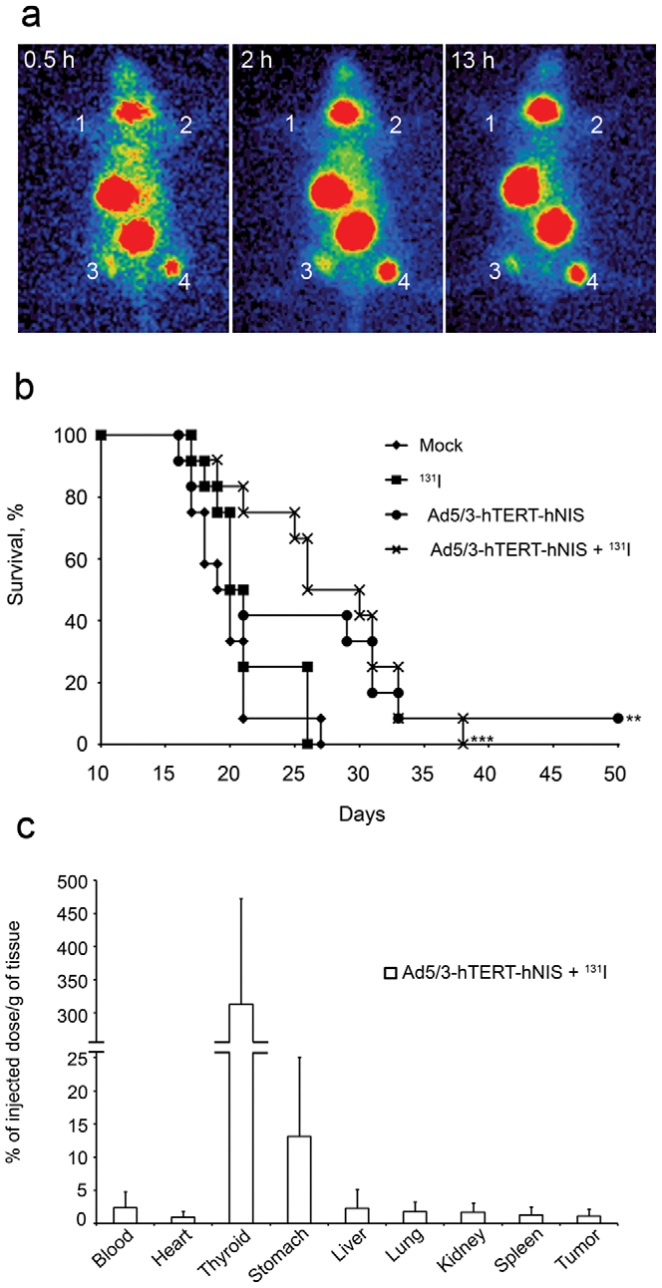
In vivo iodide uptake and efficacy. (**a**) Tumor uptake of ^123^I^−^ 0.5 h, 2 h and 13 h after i.v. administration of ^123^I^−^. The tumors were injected twice with hTERT-viruses 24 h and 48 h prior to radioiodide. 1, Mock-injected tumor; 2, Ad5/3-hTERT-Δgp19K-injected tumor; 3 and 4, Ad5/3-hTERT-hNIS-injected tumors. (**b**) Ad5/3-hTERT-hNIS significantly prolongs the survival of mice bearing intra pulmonary PC-3MM2 tumors. Mice received 5×10^10^ vp of Ad5/3-hTERT-hNIS or diluent intravenously. Next day, the mice were injected intraperitoneally with ^131^I^−^. The treatments were repeated once a week for a total of three weeks. Pairwise comparisons with the logrank test were used to compare survival curves, **p<0.01, ***p<0.001 as compared to mock-treated mice. (**c**) Biodistribution of ^131^I^−^ in mice 48 h after the first intravenous Ad5/3-hTERT-hNIS-injection and 24 h after first radioiodide-injection. Bars represent SD.

To evaluate the ability of Ad5/3-hTERT-hNIS to direct iodine uptake into prostate tumors *in vivo*, a subcutaneous tumor model was established. Each mouse had four PC-3MM2 tumors (Figure 3a). Two lowermost tumors were injected twice with Ad5/3-hTERT-hNIS and others with Ad5/3-hTERT-Δgp19K (upper right tumor) or mock (upper left tumor). To follow the kinetics of iodine accumulation into these tumors 24 h later, mice received intravenous ^123^I followed by a series of gamma-camera imaging between 0.5 h and 13 h after iodine administration. As seen in Figure 3a, Ad5/3-hTERT-hNIS allows *in vivo* accumulation of systemic radioiodine into tumors. Iodine concentration reached a plateau at 2 h post iodine-injection and remained steady until the last imaging at 13 h (Figure 3a). The variation in signal between the Ad5/3-hTERT-hNIS injected tumors is likely due to difference in tumor size as smaller tumors might have fewer blood vessels (hampering iodine access to the tumor) and may not contain as many tumor cells that allow for hNIS-expression.

^123^I accumulation was detected in the thyroid and stomach due to endogenous expression of NIS in these organs. Bladder visualization occurs due to excretion of ^123^I into urine [28]. Due to animal regulations, the experiment was ended at 13 h and did not permit us to study the effect of the treatment on tumor size.

### Efficacy of Ad5/3-hTERT-hNIS in vivo

To study the antitumor efficacy of intravenously administered Ad5/3-hTERT-hNIS with ^131^I *in vivo*, a survival experiment was set up. PC-3MM2 cells were injected into the left lung of nude/NMRI male mice (n = 12 mice/group) to mimic aggressive, castration resistant, lung-metastatic prostate cancer. A week after cell injection, the mice received 5×10^10^ vp of Ad5/3-hTERT-hNIS or diluent intravenously. The next day, mice were administered 37 MBq of therapeutic radionuclide ^131^I or PBS intraperitoneally. This treatment-cycle was repeated once a week for a total of three weeks.

The median survival in the mock group was 19.5 days (Figure 3b). Survival prolongation was seen in the Ad5/3-hTERT-hNIS+^131^I group where the median survival was 28 days. This was confirmed by statistical analyses where the p-value between mock and Ad5/3-hTERT-hNIS+^131^I was <0.001 and between ^131^I only and Ad5/3-hTERT-hNIS+^131^I it was 0.007. As expected, ^131^I only (20.5 days) did not increase survival. Although intravenous delivery of oncolytic adenovirus presents many challenges, and the model used is particularly aggressive, the overall survival of virus-only treated mice was improved over mock treatment (p = 0.04). Also, one mouse was tumor-free and healthy at the end of the experiment. Survival in the virus +^131^I group was not improved over virus –only treatment.

As radioidide might also accumulate into organs passively, or as a result of non-tumor-specific hNIS-expression, we measured the radioactivity of different organs - including tumors - from three mice from the Ad5/3-hTERT-hNIS+^131^I group, killed 24 hours after the first iodide injection (48 h after first virus injection). Due to the endogenous expression of NIS in the thyroid and stomach, about 320% and 15% of the injected dose per gram of tissue (ID/g) was concentrated in these organs, respectively. Iodide uptake into tumor was 1.3% of the injected dose (Figure 3c). The high ID/g of thyroid reflects the uneven distribution of iodine: if iodine would accumulate evenly into each organ, the ID/g would be 100%/organ.

### In vivo imaging of hNIS-expression by SPECT/CT after systemic administration of Ad5/3-hTERT-hNIS

**Figure 4:**
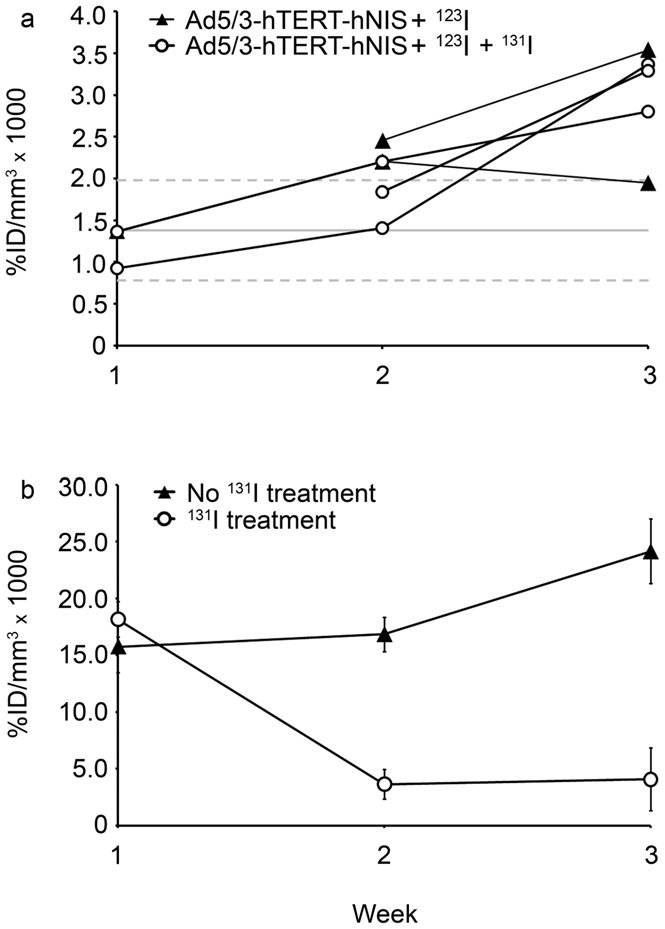
SPECT/CT is sensitive for detecting endogenous NIS- and hNIS-expression. Mice were given Ad5/3-hTERT-hNIS or diluent and ^123^I^−^ intravenously for SPECT-scanning. After the SPECT/CT-scan, mice received ^131^I^−^ or PBS intraperitoneally. The iodide uptakes were calculated weekly and normalized to the injected dose of ^123^I^−^. (**a**) Individual tumor uptake of ^123^I^−^ (the imaging isotope) in Ad5/3-hTERT-hNIS and Ad5/3-hTERT-hNIS+^131^I^−^ -treated mice. All mice received ^123^I^−^ for evaluation of iodide uptake by SPECT. Mice in the latter group also received ^131^I^−^ right after the SPECT imaging for therapeutic purposes. Solid grey line, background level in the absence of Ad5/3-hTERT-hNIS; dotted grey lines, ± SD of background. (**b**) ^123^I^−^ uptake in the thyroids. Results are expressed as %ID/mm^3^×1000 ± SD.

**Figure 5:**
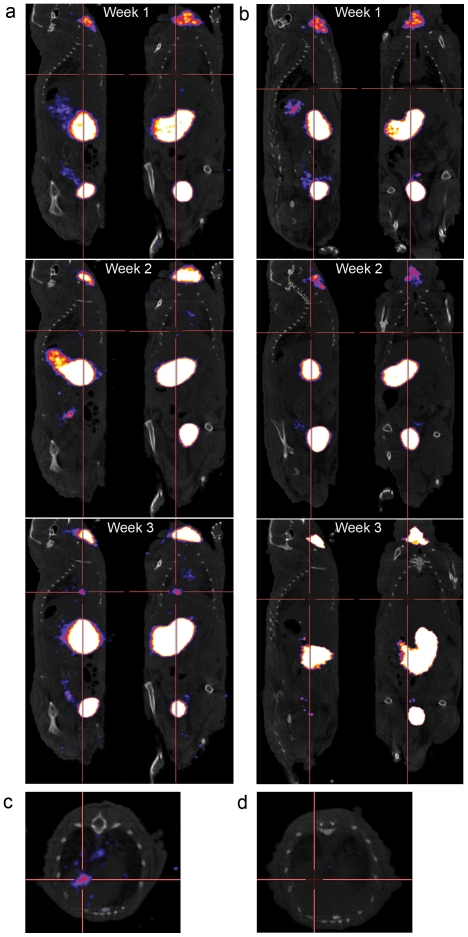
Increasing amount of ^123^I^−^ accumulates in the tumor over time showing progressive virus replication and increasing tumor hNIS-expression. (**a**) SPECT/CT image (sagittal section) showing ^123^I^−^ accumulation into tumor in the lungs of a mouse treated intravenously with Ad5/3-hTERT-hNIS and intraperitoneally with ^131^I^−^. (**b**) Control mouse treated with ^131^I^−^ only shows no ^123^I^−^ -accumulation in the lungs on the course of the treatment. (**c**) Transverse section of the lungs of the mouse shown in (**a**) shows accumulation of ^123^I^−^ into the tumor on the third treatment week. (**d**) Transverse section of lungs of the mouse shown in (**b**) shows no accumulation of ^123^I^−^ into the tumor on the third treatment week. The crossing point of red lines indicates tumor localization.

While there was a survival improvement in both virus groups over no treatment or iodine only, radioiodide did not improve survival significantly. Therefore, we performed imaging-experiments to investigate reasons for this. This permitted us also to assess the feasibility of hNIS-mediated imaging. A similar setting to the survival-experiment was employed, where mice received either vehicle, Ad5/3-hTERT-hNIS only or Ad5/3-hTERT-hNIS+^131^I. In addition, each group received 24 h after each virus/vehicle administration an intravenous bolus of ^123^I^−^ for imaging, followed by a SPECT/CT-scan and quantitation of the ^123^I^−^ signal. After the first injection, ^123^I^−^ uptake in the lungs was low and no clear tumor uptake was observed in mice treated with Ad5/3-hTERT-hNIS ± ^131^I. At this early point, ^123^I^−^ uptake to the tumors was not distinguishable from the background activity, which remained at 0.00138±0.0006%ID/mm^3^ throughout the study (Figure 4a). This result is in accordance with the observed low tumoral iodine uptake in the biodistribution assay (Figure 3c). After the second viral injection, 3/5 of the virus-treated animals showed increased ^123^I^−^ uptake in tumors, however the level of ^123^I^−^ accumulation was modest. The clearest ^123^I^−^ accumulation was reached after the third virus-injection, reaching at highest 0.0035%ID/mm^3^ (Figures 4a and 5a, c). There was no difference in tumoral ^123^I^−^ accumulation between the two Ad5/3-hTERT-hNIS treated groups, regardless if they received also the therapeutic isotope ^131^I in addition to the imaging isotope (Figure 4a). In comparison to mice that were not treated with the virus, the difference in tumor signal was clear (Figure 5). The ^123^I^−^ uptake-results are summarized in Table 1.

**Table 1:** Number of mice positive for uptake of the imaging isotope ^123^I at the tumor.

	Day		
Group	1	7	14
Ad5/3-hTERT-hNIS alone	0 (2)	2 (2)	1 (2)
Ad5/3-hTERT-hNIS+^131^I	1 (3)	1 (3)	3 (3)
^131^I alone	0 (2)	0 (2)	0 (2)
Mock	0 (2)	0 (1)	-

The total number of mice analyzed at each time point is given in parentheses. Day indicates the day after first intravenous virus injection. -, not analyzed.

These data suggest that intravenous injections of Ad5/3-hTERT-hNIS allow tumor transduction, virus replication, hNIS-expression and radioiodide accumulation to such degree that it can be detected with SPECT/CT. The signal volume detected by SPECT was rather small and the mice nevertheless died, which led us to perform autopsies at the end of the therapeutic experiment. There was a discrepancy between actual tumor size seen in autopsy and the radioiodide accumulation dependent SPECT signal: tumors seemed larger than what was observed by SPECT signal. Possible explanations for this include incomplete penetration of the tumor by either virus or by ^123^I^−^.

All animals showed normal endogenous NIS expression in the thyroid, stomach, salivary and lachrymal glands. With the exception of the thyroid in ^131^I-treated animals, iodide accumulation into tissues expressing endogenous NIS was not affected. As the thyroid was not protected from radioiodide uptake in this experimental design, animals treated with ^131^I showed diminished thyroid activity in the second and third SPECT scan, as expected (Figure 4b). In addition, some mice showed physiological ^123^I^−^ uptake in the esophagus, which was easily differentiated from the tumoral uptake using the anatomical information of the co-registered CT scan (Video S1).

Dose ratios (approximated as uptake ratios) thyroid∶blood∶kidney∶tumor were approximately 1000∶1.4∶1.0∶0.8 (Figure 3c). If the thyroid is protected or thyroid ablation accepted, the dose limiting normal tissue would be the red bone marrow. Assuming that the blood dose can be used as a surrogate for the bone marrow dose (i.e., assuming no active iodine trapping from blood to bone marrow and cross-dose omitted), the typical highest allowed blood dose (2 Gy/treatment cycle) would contribute 1.1 Gy to the tumor which is perhaps unlikely to result in therapeutic effect. These figures can be compared with the standard human kinetics applied for the dosimetry of internal radiopharmaceuticals (sodium iodide, oral administration, [29]) assuming 25% uptake of iodine in the thyroid at 24 h. These calculations result in thyroid∶bone marrow∶kidney ratios of 1000∶2.4∶1.05, respectively, which are in the same range as the mouse figures reported here.

After intravenous adenovirus injections, a large proportion of the dose is taken up by the liver [30]. Therefore, we analyzed the hepatic radioiodide signal which would reflect “leaky” expression of virally-encoded hNIS in a non-target organ, keeping in mind that our virus-design predicts hNIS-expression mostly in cells that allow virus replication. Only two out of five virus-treated animals showed any degree of liver accumulation of ^123^I^−^ after the first virus-treatment. This is in dramatic contrast to non-regulated expression of transgenes following intravenous adenovirus injection [31]. After subsequent virus injections during the next two weeks, no iodide uptake was detected (Figure S1) and the signal difference compared to non-virus treated mice was insignificant (p = 0.24). This demonstrated that hNIS-expression from Ad5/3-hTERT-hNIS was mostly restricted to tumor cells and leaky expression in normal tissues was minimal (Video S1).

## Discussion

Several tissue-specific promoters have been exploited for targeting oncolytic adenoviruses and restricting their gene expression to target tumors [32]. While stringent tissue-specificity has been reported, the drawback of this approach lies in the selectivity of replication in only one or a few tumor types. However, hTERT-expression is ubiquitous in most human tumors while limited in normal somatic cells [14], making it an attractive promoter for specific adenoviral replication in human tumors. Importantly, good safety data has been obtained in a human trial featuring an hTERT-controlled oncolytic adenovirus [19]. hTERT is expressed in stem cells. However, since stem cell toxicity is not an established feature of wild type adenovirus infection [33], we can assume that stem cells have other ways to protect themselves against virus as suggested by preliminary experiments performed in normal breast stem cells [16]. Although this study used Ad5/3-hTERT-hNIS to treat castration resistant prostate cancer, Ad5/3-hTERT-hNIS could also be used to kill hTERT-positive cancer cells of different origins.

The rationale for using hNIS in cancer gene therapy has already been established [7], [8], [9], [34]. In our virus, replication-induced hNIS–expression from the adenoviral E3-region couples radiographic imaging via hNIS to viral replication kinetics. We found it feasible to assess these aspects by using SPECT. Moreover, hNIS-expression by Ad5/3-hTERT-hNIS could allow dual-modality destruction of infected tumors: therapeutic radioiodide-treatment and oncolysis. While the same cell does not need to be killed twice, the biggest utility might come from the two different bystander effects. Oncolysis can amplify the input virus dose and in theory the process continues as long as permissive cells exist. However, tumors are complex, heterogeneous and contain areas impassable for the virus, including stromal and necrotic areas. Radiation, however, could exert effects on, over and through such areas. Also, some cells are or become resistant to radiation or oncolysis [35], [36]. In this context, the strong synergy between oncolytic adenoviruses and radiation could be useful [4], [37] as suggested in previous reports [7], [9], [38], [39]. However, since there was no significant difference to the virus only in our experiment, construct design issues may play a role in the relative impact of oncolysis versus iodine mediated killing. In the context of optimized viral replication, a large sample size or an immune competent organism (where the efficacy of oncolysis could be less prominent) may be needed for demonstrating if there is any benefit of the combination in terms of efficacy.

Autopsies performed at the end of imaging experiment suggested that tumor masses were larger than what were observed by SPECT-imaging. Thus, the virus might not have been simultaneously replicating in the entire tumor and only the part of the tumor that sustains active replication at the time of imaging would be positive for hNIS-expression – uninfected or dead tissue would not be detected. The heterogeneity of the tumor mass might also contribute: although hTERT-activation would be detected in the parental tumor cells, the rapidly growing tumor bulk could allow for variation in hTERT-promoter activity, and thus in hNIS expression, due to mutations and inactivations of regulatory factors like the E-box motifs, which have been suggested to be the major determinants of hTERT-expression [40], [41]. Furthermore, intratumoral barriers including stromal, necrotic, hyperbaric and hypoxic areas are likely to hamper the penetration of the virus to the entire tumor resulting in an uneven distribution of hNIS-expression. Incomplete tumor transduction due to polarized expression of viral receptors could also play a role [42]. Finally, as predicted by the mechanism of the virus, only tumor areas through which the virus replication front is proceeding would be expected to be positive for hNIS. Areas in which virus replication has already occurred, would not be hNIS-positive, as cells that allowed for replication would have been lysed. Alternatively, oncolytic treatment might render tumor vasculature leaky or create local hypobaric areas resulting in areas of hNIS-independent iodine influx. This possibility could not be excluded since treatment with control virus was not assessed in the systemic approach. Also, in some tumors, only a small proportion of cells are malignant [43], [44], while the rest are murine-derived stromal (not permissive for adenovirus replication) and the latter would remain after oncolysis.

Visualization of the thyroid confirmed the utility of SPECT imaging in the context of radioiodide therapy. Notably, all mice treated with ^131^I showed diminished ^123^I^−^ uptake by the thyroid during 2^nd^ and 3^rd^ week of the treatment. There are, however, differences regarding hNIS-expression as a transgene or as a regulator of the endocrine system. Importantly, extrathyroidal tissues expressing hNIS are unable to organify iodide and their hNIS-expression is not regulated by thyroid stimulating hormone (TSH) [45]. In contrast, hNIS-expression in the thyroid is directly regulated by serum iodine concentration and TSH [46]. TSH expression and secretion, in turn, are regulated by thyroxin and triiodothyronine, and their relationship is inversely correlated [47]. Thus, the thyroid could be protected from ^131^I either by iodine- or thyroxin-supplementation prior to therapy. The latter has been successfully used in glioma treatment with a lentiviral vector expressing hNIS [48]. The study reported a lack of thyroidal NIS-expression and function after a thyroxin supplemented diet whereas hNIS-transduced tumor tissue could be simultaneously visualized.

Excess iodine decreases NIS mRNA and protein expression [49], reduces radioiodide accumulation into the thyroid and can protect the thyroid for 24 h when administered up to two hours prior to ^131^I [50]. If the same timing of Ad5/3-hTERT-hNIS and ^131^I would apply in a clinical setting, extra-iodine administered 4–6 hours after virus injection would saturate the thyroid without hampering transgene-mediated transportation of radioiodide into the tumor after ^131^I administration (eg. at 24 hours as in our experiments). However, lack of organification of radioiodide into cancer tissues is also a drawback as it reduces accumulation and subsequent dose to the tumor. A rapid radioiodide efflux from tumor cells has been associated with exogenous hNIS-expression [51] and it is clear that this issue is critical in our approach. Another problem may arise from viral oncolysis resulting in the death of the hNIS-expressing cells that limits the duration of the hNIS-expression window. The “timed” expression system used in Ad5/3-hTERT-hNIS, where transgene-expression starts at circa 8 h after infection (and thus closer to oncolysis), may limit the window further. In this regard, a ubiquitous CMV-promoter with high early activity might result in higher hNIS-expression levels [52], [53]. When adenoviral vectors are delivered systemically, they are rapidly taken up by liver Kuppfer cells (KC) [30]. The phenomenon is dose dependent and saturation occurs after high intravenous doses [54] leading to efficient transduction of hepatocytes and other tissues. Adenoviral entry into hepatocytes together with KC activation can result in an immune response that is suggested to be the major determinant of adenovirus induced toxicity [55]. However, if the injected doses are small enough to avoid acute hepatotoxicity, but sufficient to saturate KC, transgene expression in hepatocytes occurs. This provokes a second, adenovirus-protein -specific immunoresponse [56]. Thus, the use of tumor-specific promoters to restrict expression of adenoviral proteins to tumors is important from a safety perspective. Indeed, previous work has shown that hTERT-regulated expression of *E1A* in an oncolytic adenovirus did not induce liver toxicity [57]. Accordingly, minimal hepatic hNIS-expression was seen in our study.

Human adenovirus is fairly human-specific and thus mice are poor models for studying toxicity. Ultimately, human trials may be needed to evaluate the feasibility and safety of intravenous injection at doses compatible with efficacy. Oncolytic adenoviruses might currently be the best administered intratumorally or intracavitary, perhaps in combination with intravascular delivery. Nevertheless, there are some tantalizing cases suggesting efficacy even after intravenous delivery alone [3], [58]. However, immunogenicity of adenoviruses is an issue with regard to systemic re-administration [59].

In conclusion, Ad5/3-hTERT-hNIS was found to have anti-tumor activity *in vitro*, and *in vivo* it extended the survival of mice. Intriguingly, SPECT/CT imaging suggested that hNIS-expression could be detected only in a proportion of the tumor mass, which is likely due to incomplete penetration after systemic administration. Accordingly, combination therapy was not more effective than virus alone. However, an increasing level of hNIS-expression during therapy was detected using SPECT/CT, demonstrating the utility of the method for dynamic imaging of radioiodide therapy in animals with complex orthotopic human cancer xenografts. Before considering the possible clinical translation of the combination therapy to humans, further work is needed for improving tumoral hNIS expression levels and consequently ^131^I-targeting efficiency [52].

## Materials and Methods

### Cell lines

Castration resistant prostate cancer cell lines 22Rv1, PC-3 and DU-145 and human embryonic kidney epithelial cell line 293 were purchased from American Type Culture Collection (ATCC, Manassas, VA). PC-3MM2 cells are a metastatic, castration resistant subline of PC-3 (courtesy of Isaiah J. Fidler, M. D. Anderson Cancer Center). 911 cells were obtained from Dr. Alex J. van der Eb (University of Leiden, The Netherlands). All cell lines were cultured as previously described [7]. PC-3 and DU-145 cells have been confirmed to have hTERT-expression [60].

### Viral constructs

Wild type adenovirus serotype 5 (Ad5 WT) was purchased from ATCC. Ad5/3luc1 has been described earlier [22].

### Cloning

hTERT-fragment was amplified by PCR from pBT255 (Courtesy of Izumi Horikawa, National Cancer Institute) to create a shuttle vector for E1-region. The following primers were used to result in 361 bp sized amplification product: forward primer, 5′-TTA GCG GCC GCA GAA CAT TTC TCT ATC GA-3′ and reverse primer, 5′-ACC GGA ATG CCA AGC TTA CTT AGA T -3′. NotI-XhoI- digested hTERT-fragment was inserted into NotI-XhoI -digested and calf intestinal alkaline phosphatase (CIAP) -treated pSE1Amp^R^ (constructed previously in the laboratory) to obtain phTERTE1Amp^R^. Next, both PmeI -linearized phTERTE1Amp^R^ and circular pAdEasy-1.5/3-Δ24 [61] were electroporated into BJ5183 cells for homologous recombination resulting in pAdEasy-hTERT-E1-5/3. DNA from selected recombinant clones was electroporated into DH5α cells for DNA production and further analysis.

To obtain phTERT-E1-5/3-hNIS and phTERT-E1-5/3-Δgp19K, SrfI-linearized pAdEasy-hTERT-E1-5/3 and FspI –linearized pTHSN-hNIS or FspI –linearized pTHSN-Δgp19K [7] were electroporated into BJ5183 cells for homologous recombination. The DNA from selected recombinant clones was electroporated into DH5α cells for DNA-production and further analysis. phTERT-E1-5/3-hNIS and phTERT-E1-5/3-Δgp19K were linearized and transfected into 911 cells using Superfect (Qiagen, Hilden, Germany). The viruses were plaque-purified and propagation was performed on A549 cells followed by standard CsCl purification to obtain a concentration of 4.8×10^11^ viral particles/ml (vp/ml) and 1.8×10^10^ plaque forming units/ml (pfu/ml) for Ad5/3-hTERT-hNIS and 5×10^11^ vp/ml and 5.1×10^10^ pfu/ml for Ad5/3-hTERT-Δgp19K.

### Reverse transcription –PCR (RT-PCR)

Prostate cancer cells were infected with 10 vp/cell of Ad5/3-hTERT-hNIS or Ad5/3-hTERT-Δgp19K for 2 h in 37°C. 24 h and 48 h after infection total RNA was isolated (RNeasy Kit, Qiagen). The samples were treated with DNase before subjected to RT-PCR. 350 ng of RNA was used for each reaction. Amplification was done using OneStep Reverse Transcription –PCR Kit (Qiagen) with 35 cycles and annealing at 54°C. The hNIS-PCR product size (453 bp) was obtained using hNIS-specific primers (forward 5′-CTTCTGAACTCGGTCCTCAC-3′ and reverse 5′-TCCAGAATGTATAGCGGCTC-3′). For ß-actin, (482 bp product size) was obtained using for human ß-actin-specific primers (forward 5′-CGAGGCCCAGAGCAAGACA-3′ and reverse 5′-CACAGCTTCTCCTTAATGTCACG-3′).

### Iodine uptake in vitro

1×10^5^ PC-3, PC-3MM2, DU-145 and 22Rv1 cells/well were plated, incubated overnight, and next day infected with 10 vp/cell of Ad5/3-hTERT-hNIS or Ad5/3-hTERT-Δgp19K for two hours. 24 h or 48 h later the cells were washed with PBS and [^125^I]NaI (MAP Medical Technologies Oy, Finland) was added for 20 min at RT. Cells were washed twice with PBS and 300 µl/well of Cell Culture Lysis Reagent (Promega, Fitchburg, WI) was added. Radioactivity was quantified as described in [7].

### Cell killing assays

10,000 cells/well were seeded on 96-well plates, incubated overnight and next day infected with Ad5/3-hTERT-hNIS, Ad5/3-hTERT-Δgp19K, Ad5/3luc1 or Ad5 wild type (0.01–100 vp/cell) for 2 h. The viability of the cells was assessed on day 6 (PC-3MM2), day 7 (PC-3 and DU-145) or day 9 (22Rv1) using the MTS-assay (CellTiter 96 Aqueous One Solution cell proliferation assay, Promega). The cell survival after infection was followed daily with internal controls and the stopped when an almost complete cell killing was seen with the fastest virus [24].

### Iodine uptake in vivo

The protocol was identical as described in [7]. NMRI/nude mice (Taconic, Ejby, Denmark) were injected with 7×10^8^ vp of Ad5/3-hTERT-hNIS, Ad5/3-hTERT-Δgp19K or saline.

### In vivo survival and biodistribution

NMRI/nude mice (n = 15/group) were purchased from Taconic. 1.5×10^6^ PC-3MM2 cells were inoculated into the left lung of the mice under medetomidine-ketamine anesthesia (day 0) [4], [7]. On day 7, mice were randomized into four groups; mock, ^131^I alone, Ad5/3-hTERT-hNIS alone or ^131^I+Ad5/3-hTERT-hNIS and received 5×10^10^ vp of Ad5/3-hTERT-hNIS or DMEM intravenously. The next day, animals were injected with 36±5 MBq of [^131^I]NaI (MAP Medical Technologies) in 1×PBS or vehicle intraperitoneally. Three animals from each group were sacrificed 24 hours after the first ^131^I-treatment for the biodistribution study where radioactivity was measured with an automated gamma-counter (Perkin Elmer Wizard 3, Perkin Elmer, Waltham, MA). Results are expressed as percentage from the initial iodine-dose/g of tissue. 12 mice/group continued in the survival experiment and the same doses were repeated once a week for 3 weeks.

### hNIS-expression in vivo by SPECT/CT

NMRI/nude male mice (Harlan, Indianapolis, IN) were treated identical to the survival-experiment. The animals were scanned with SPECT/CT a day after each intravenous Ad5/3-hTERT-hNIS or DMEM injection, three times in total. The ^131^I (32±6 MBq) or PBS-injections were given after the SPECT/CT-scan.

18±2 MBq of sterile [^123^I]NaI (MAP Medical Technologies) was injected intravenously under isoflurane-anaesthesia for SPECT-imaging (that was performed 2 h after ^123^I injections). SPECT/CT was acquired using multi-pinhole nanoSPECT/CT-system with four heads (Bioscan, Washington DC). SPECT-acquisition collected 30 000 counts per projection (16-projections) and the scan-time was 20–25 min. After the third scan mice were sacrificed and the tumors were harvested. All images were reconstructed with MEDISO-software and analyzed using In Vivo Scope-software (both Medical Imaging Systems, Kingsbury, England). Voxel-guided regions of interest (ROI) were drawn using the fused SPECT/CT-images and the results are expressed as percentage of radioactivity in the ROI from the injected activity (at time of the SPECT-scan), divided by the volume of the ROI (in mm^3^).

### Statistical analysis

Student's t-tests were performed to analyze *in vitro* iodide uptake and cell killing using Microsoft Excel (Microsoft Corporation, WA). Survival analyses were conducted by plotting Kaplan-Meier curves, and the comparisons between groups were done pairwise by the log-rank procedure with PASW statistics 17.0 (SPSS Inc., Chicago, IL). For all analyses, a two-tailed p-value<0.05 was deemed statistically significant.

### Ethics statement

The animal experiments were approved by the National Committee for Animal Experimentation in Finland (State Provincial Office of Southern Finland), permit number ESLH-2008-00590-Ym-23.

## Supporting Information

Figure S1 **hNIS-expression from Ad5/3-hTERT-hNIS is restricted to tumor cells.** A mouse treated with Ad5/3-hTERT-hNIS shows minimal liver ^123^I^−^ accumulation after the first intravenous injection of the virus (week 1). The next treatments (weeks 2 and 3) did not result in hepatic uptake.
(TIF)

Video S1 **The video shows tumor-specific hNIS-expression on the third treatment week in the lung of a mouse treated with Ad5/3-hTERT-hNIS and ^131^I^−^.** Some physiological uptake is seen in esophagus, thyroid and salivary glands.
(GIF)
