# Supplementary figures and images for: SPECT/CT Imaging of hNIS -Expression after Intravenous Delivery of an Oncolytic Adenovirus and 131I
Source: PLoS One. 2012 Mar 7;7(3):e32871. doi: 10.1371/journal.pone.0032871 (PMC3296755; doi:10.1371/journal.pone.0032871)

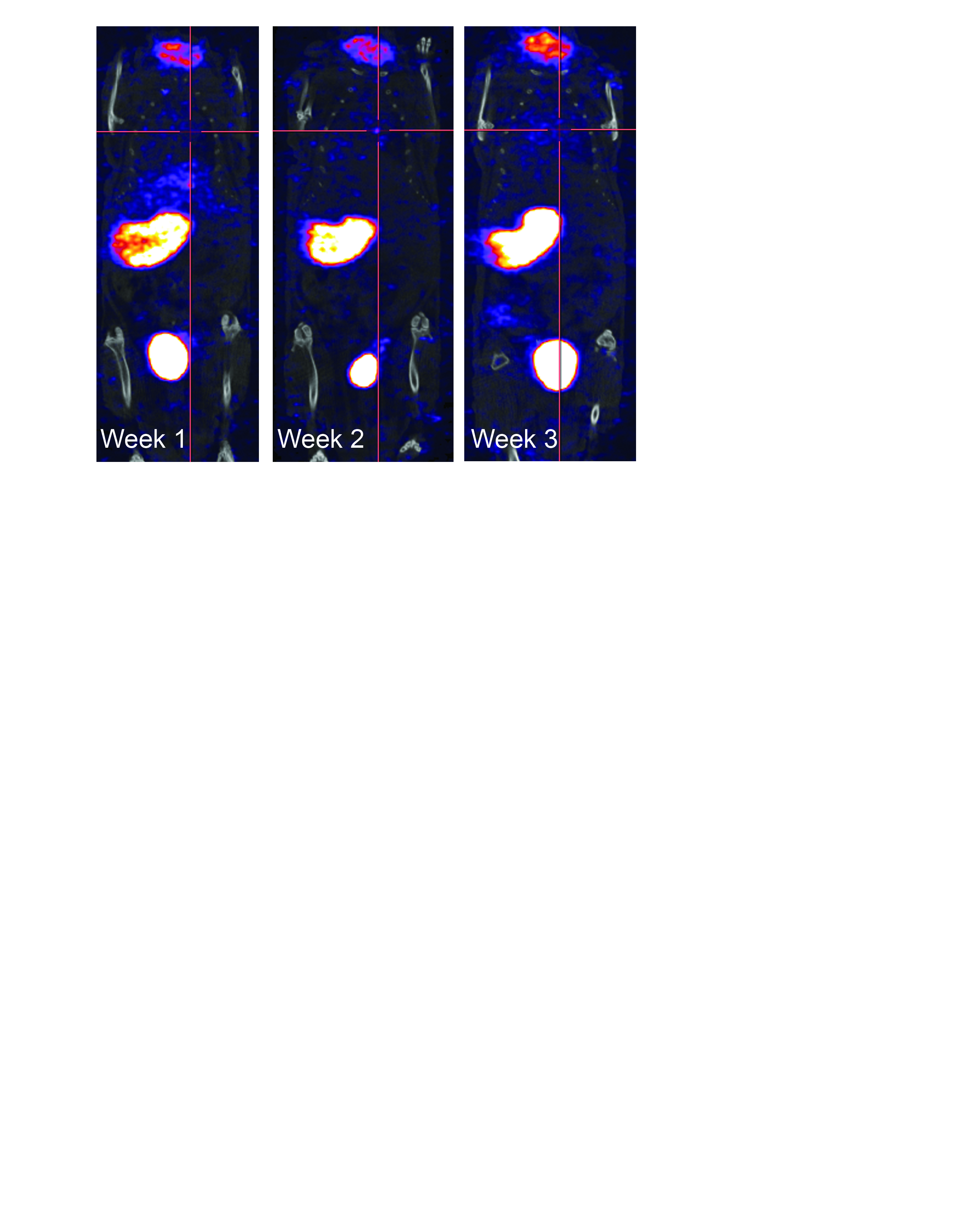

Supplement: Figure S1 — hNIS-expression from Ad5/3-hTERT-hNIS is restricted to tumor cells. A mouse treated with Ad5/3-hTERT-hNIS shows minimal liver 123I− accumulation after the first intravenous injection of the virus (week 1). The next treatments (weeks 2 and 3) did not result in hepatic uptake. (TIF) [file pone.0032871.s001.tif]

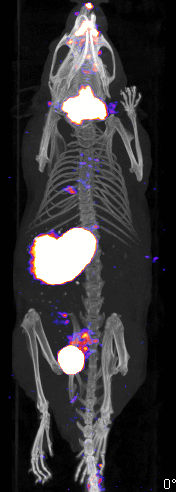

Supplement: Video S1 — The video shows tumor-specific hNIS-expression on the third treatment week in the lung of a mouse treated with Ad5/3-hTERT-hNIS and 131I−. Some physiological uptake is seen in esophagus, thyroid and salivary glands. (GIF) [file pone.0032871.s002.gif]
